# Reliability of Ultrasonography to Assess Spinal Compression During Heavy Load Carriage

**DOI:** 10.1002/jsp2.70137

**Published:** 2026-02-10

**Authors:** Sherrilyn Walters, Ben Hoffman, Celeste E. Coltman, Lester Walters, Muneeb Iqbal, Dean E. Mills

**Affiliations:** ^1^ School of Health and Medical Sciences University of Southern Queensland Ipswich Queensland Australia; ^2^ Martial Arts Research and Testing Laboratory Toowoomba Queensland Australia; ^3^ School of Medicine and Dentistry Griffith University Gold Coast Queensland Australia; ^4^ Centre for Health Research, Institute for Resilient Regions University of Southern Queensland Ipswich Queensland Australia; ^5^ Faculty of Health University of Canberra Research Institute for Sport and Exercise, University of Canberra Bruce Australian Capital Territory Australia

**Keywords:** axial loading, back injury, back pain, heavy load carriage, lower back, lumbar vertebrae, posture, spinal compression, spine, ultrasonography

## Abstract

**Background:**

Back pain and spinal injury are leading contributors to premature retirement, particularly in physically demanding occupations. Direct and practical methods of spinal assessment are needed to evaluate interventions aimed at reducing spinal loading and injury risk. Ultrasonography has been reliably used to estimate spinal compression via intervertebral disc height, but its reliability for measuring inter‐transverse process distances under load has not been established.

**Methods:**

Eleven healthy adults underwent ultrasonographic measurement of inter‐transverse process distances at each lumbar level (L1–L5), and the total lumbar distance under four loading conditions: (1) immediately on standing while unloaded, (2) after 15 min of unloaded standing, (3) after 15 min of standing loaded with a 25 kg weighted vest, and (4) after 30 min of loaded standing. These procedures were repeated after 1–7 days. Inter‐rater, within‐visit, and between‐visit reliability were assessed using intraclass correlation coefficients (ICCs) and coefficients of variation (CV). Bland–Altman plots were used to assess agreement. A one‐way analysis of variance was used to determine the effects of each loading condition on inter‐transverse process distances.

**Results:**

Inter‐rater, within‐visit, and between‐visit reliability was good to excellent with ICCs between 0.81 and 0.99 and CVs between 5.24% and 13.0% for all measurements. Inter‐transverse process distances were reduced at L2/3 (*p* = 0.007), L3/4 (*p* = 0.006), and across the total lumbar distance (*p* = 0.02) following 15 and 30 min of loaded standing.

**Conclusion:**

Ultrasonography is a reliable, low‐cost method for quantifying changes in lumbar spine geometry during loaded standing. This technique may have value in occupational and clinical settings for assessing spinal compression in response to mechanical load.

## Graphical abstract

This study evaluated the reliability of ultrasonography to measure inter‐transverse process distances in the lumbar spine during standing load carriage. Eleven healthy adults underwent ultrasound assessments during unloaded and loaded standing conditions. Inter‐rater and test‐retest reliability were good to excellent (ICCs 0.81–0.99). Loaded standing reduced inter‐transverse process distances at L2/3, L3/4, and total lumbar inter‐transverse process distance. Ultrasonography is a reliable, low‐cost method to quantify spinal compression during load, with potential occupational and clinical applications.

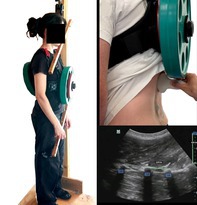

## 1 Introduction

Lower back pain is the primary cause of disability globally, with over half a billion cases worldwide in 2020 [1]. Occupational activities including lifting, manual handling, and heavy load carriage are moderately associated with intervertebral disc disruption, characterized by reduced intervertebral distance and other structural changes to the lumbar vertebral discs [2, 3, 4, 5]. Furthermore, back pain and injury are leading chronic conditions associated with premature retirement, particularly in active occupations such as Defense [5, 6, 7]. Several forms of exercise may help to address the prevalence and severity of lower back pain. Intense physical activity has been linked to reductions in lower back pain, and exercise of the abdominal, respiratory and pelvic floor musculature may contribute to unloading and support of the spine through the generation and control of intra‐abdominal pressure [8, 9]. In order to objectively assess the preventative and/or rehabilitative effects of exercise interventions on the spine, direct and practical methods of assessing changes to the vertebra and intervertebral disc distance are required.

Magnetic resonance imaging (MRI) is considered the gold standard for assessing intervertebral disc morphology due to superior soft tissue contrast resolution compared with other imaging modalities such as radiography and computed tomography [10]. However, MRI is costly and impractical when assessing the response of the spine to occupational tasks, including heavy load carriage, which requires the participant to be standing and loaded with a mass. In contrast, ultrasonography offers a portable, cost‐effective, and versatile alternative to spinal assessment that complements MRI by overcoming these limitations and allowing real‐time measurements during dynamic tasks [11, 12, 13, 14, 15, 16, 17, 18]. Previous studies have established good to excellent reliability and accuracy of ultrasonography for measuring lumbar inter‐segmental distances and have demonstrated its effectiveness in detecting spinal compression under various static and controlled conditions [11, 13, 17]. Moreover, research has found that ultrasonography can be used to detect significant load‐related changes in lumbar spine distances across specific vertebral levels, highlighting its potential for real‐time monitoring in practical settings [13, 17, 18, 19].

However, the inter‐rater and between‐visit reliability of ultrasonography for assessing intervertebral disc distance during occupationally relevant load carriage has not been established. Establishing this reliability is essential for the use of ultrasonography to assess spinal responses and evaluate exercise interventions in ecologically relevant conditions, which would not be feasible with MRI. Real‐time ultrasound monitoring could allow early detection of spinal loading abnormalities, guide interventions, and provide biofeedback during rehabilitation or preventative training. In research, it may facilitate longitudinal tracking of spinal health in occupational populations and inform preventative strategies to reduce lower back pain. However, physiological factors such as diurnal variation due to fluid redistribution must be considered when interpreting ultrasonographic measurements [12, 20, 21]. While standardized timing and pre‐imaging protocols can minimize this effect, some variability may persist even under controlled conditions.

Accordingly, we aimed to (1) assess the inter‐rater and within‐ and between‐visit reliability of ultrasonography to quantify lumbar inter‐transverse process distances reflective of spinal compression during unloaded standing and after heavy load carriage; and (2) identify the presence and magnitude of spinal compression during heavy load carriage using ultrasonography.

## 2 Materials and Methods

### 2.1 Participants

Eleven healthy adults (five male/six female; age: 33 ± 5 years; height: 1.55 ± 0.51 m; body mass: 74.9 ± 18.3 kg) participated in the study following screening [22] and completion of a customized medical history questionnaire. Participants were excluded if they were under 18 or over 45 years of age; had a body mass index under 18 or over 35 kg/m^2^; had a history of orthopedic diseases or dysfunction; or a history of lower back, neck and/or hip pain. Participants refrained from strenuous exercise in the 24 h before testing due to the potential effect of prior exercise on spinal compression. All participants provided written, informed consent, and ethics approval was obtained from the University Research Ethics Committee (H22REA120).

### 2.2 Experimental Design

**FIGURE 1:**
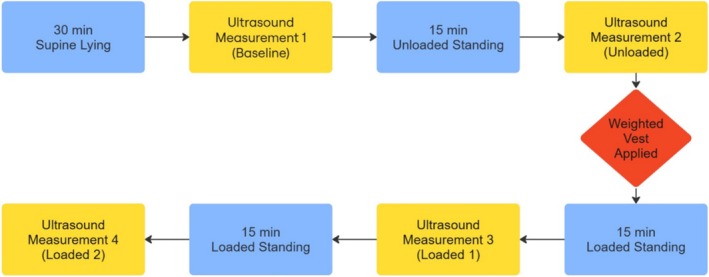
Flowchart showing the sequence of body positions and ultrasound measurements.

**FIGURE 2:**
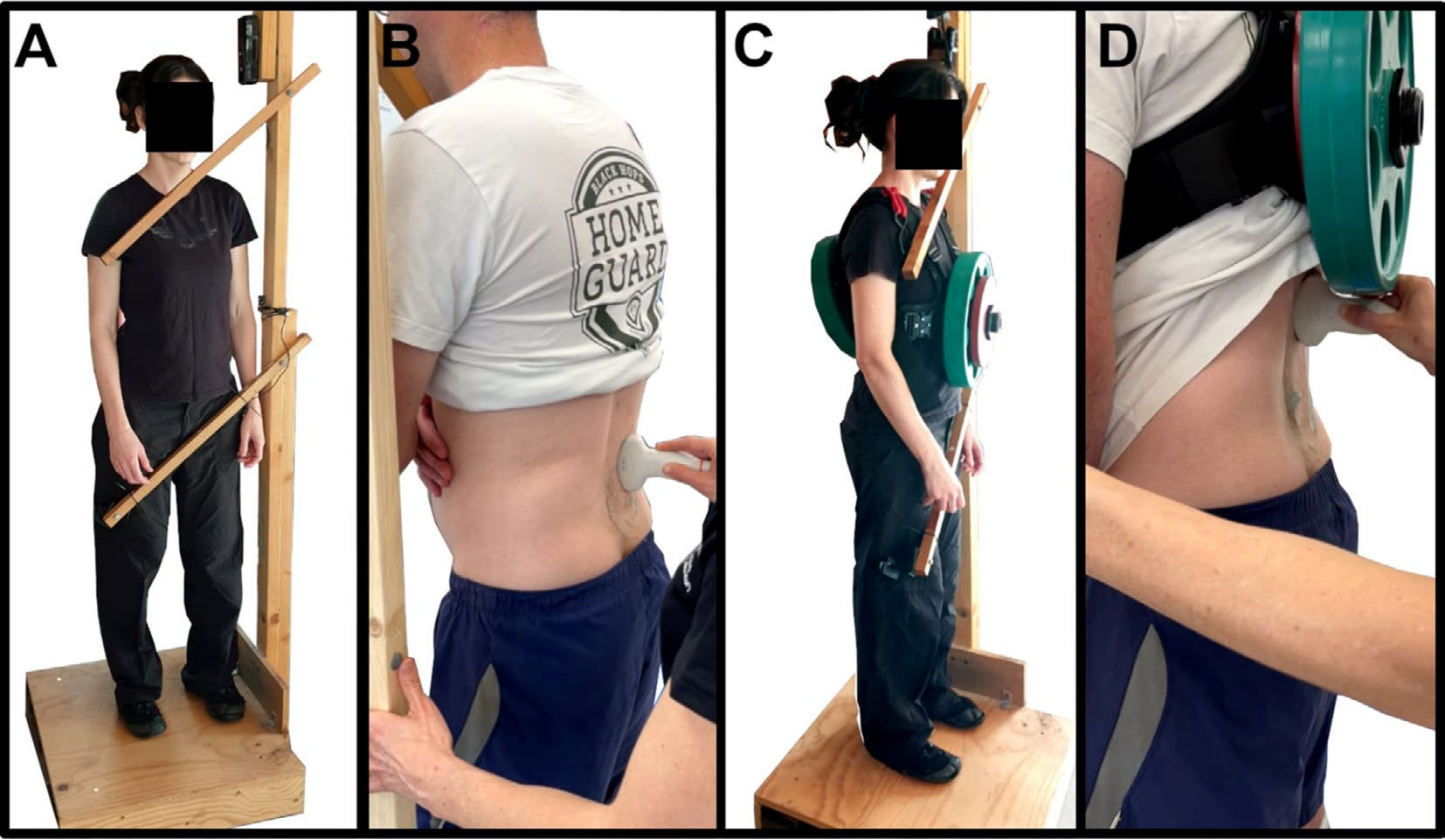
(A) Unloaded standing showing platform and position of bars. (B) Ultrasound measurement during unloaded standing. (C) Loaded standing showing platform and position of bars. (D) Ultrasound measurement during loaded standing.

Participants attended the laboratory on two separate days, with each visit separated by a minimum of 1 and a maximum of 7 days. To minimize the effects of diurnal variation in spinal height due to fluid redistribution within the intervertebral discs, visits were scheduled at the same time of day for both testing sessions [12]. During visit 1, height (custom‐made wall‐mounted stadiometer) and body mass (Tanita Ultimate Scale 2000; Tanita, Tokyo, Japan) were measured. Subsequently, participants rested in a supine position for 30 min to allow equilibration of intervertebral disc height prior to imaging [23]. Participants then assumed a standing position, and the transverse processes of the lumbar vertebrae were imaged via ultrasound immediately after standing (Baseline). Participants continued to stand for 15 min, and the ultrasound imaging was repeated (Unloaded). A weighted vest (Ez Vest Max V2; Kensui Fitness, Sheridan, WY, USA) loaded with 25 kg (12.5 kg located anteriorly and 12.5 kg located posteriorly) was then placed onto the participant's torso. This weight is representative of loads carried in physically demanding occupations such as the military and emergency services [24]. Ultrasound measurements were repeated after 15 (Loaded 1) and 30 (Loaded 2) min of standing (Figure 1). A platform was constructed with two wooden bars adjusted to contact participants' hip/thigh, and shoulder/chest while participants were standing in a comfortable position (Figure 2). During the first set of measurements, after the bars were positioned as described and secured in place, the angle of each bar was measured and recorded, and used during subsequent measurements to ensure a consistent body position throughout. During visit 2, all procedures were repeated except for body mass and height measurements.

### 2.3 Ultrasound

A curvilinear probe (C5‐2s 50 mm Convex Probe; Mindray, Shenzhen, China) was used with the ultrasound system (M7; Mindray, Shenzhen, China). The probe was positioned longitudinally on the participant's skin, using a water‐based gel (Aquasonic Ultrasound Gel; Parker Laboratories, Fairfield, NJ, USA) as a coupling agent, with the orientation marker on the probe pointed cephalad. The tips of the transverse processes of the lumbar vertebra were selected as an anatomic landmark as they have been previously identified repeatedly and consistently by raters [12]. The investigator (rater 1) first identified the sacrum and spinus process of L5. The probe was then slowly moved ~3 cm laterally to the right of midline to locate the transverse processes. The investigator then slowly moved the probe cranially along the transverse processes from L5 to L1, and then back to L5, ensuring that the tip of each transverse process was visualized on both passes. During this procedure, video footage was recorded from the ultrasound system for subsequent offline analysis. This procedure was repeated during visit 2.

### 2.4 Training and Blinding of Raters

Both raters involved in image analysis underwent comprehensive training before data collection and analysis, including instruction by senior researchers experienced in musculoskeletal ultrasonography and lumbar spinal anatomy. This training covered identification of transverse process landmarks and standardized measurement techniques. The raters undertook side‐by‐side analysis and discussion of sample images until both raters achieved consistent measurements. For data collection and analysis, rater 1 conducted measurements with knowledge of participants and loading conditions but remained blinded to rater 2's analysis. Rater 1 also randomized the anonymized image files prior to analysis by rater 2. Rater 2 was blinded to participants information, loading conditions, and rater 1's results. The use of trained independent raters with structured blinding procedures aligns with best practices for reliability studies in musculoskeletal imaging and has been shown to enhance reproducibility of ultrasound‐based spinal measures [12, 15, 18].

### 2.5 Ultrasound Image Analysis

**FIGURE 3:**
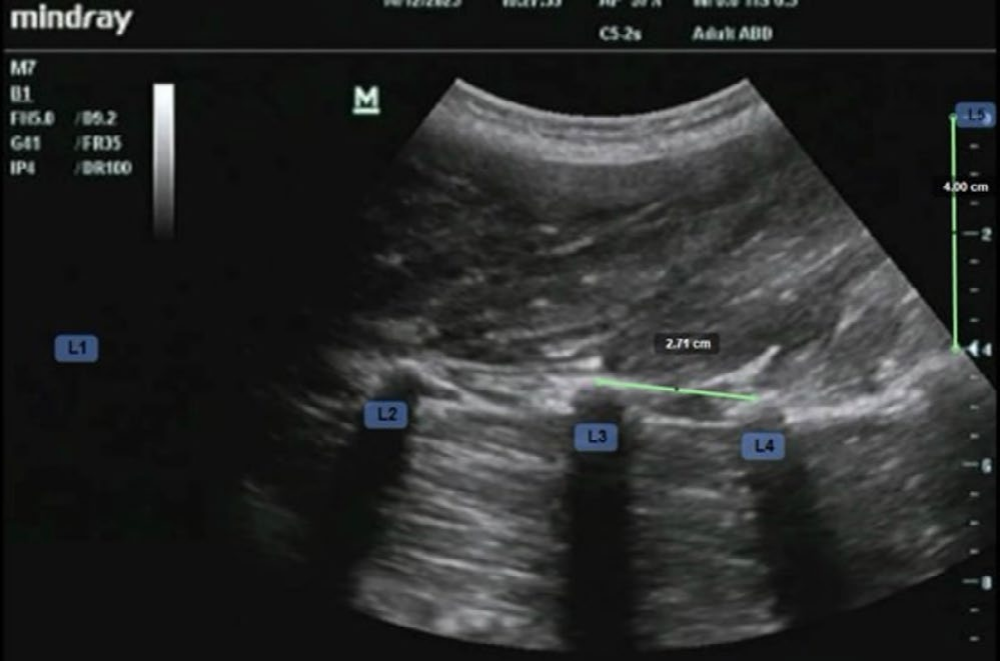
An example ultrasound image showing the measured distance between the L3 and L4 transverse processes (L3/4).

Video footage was analyzed offline using Kinovea software (version 0.8.15, www.kinovea.org), which was chosen for ease of use in offline video and still image analysis, and prior use in the literature [25, 26]. Still images were captured at each spinal level, ensuring that the tips of the respective pair of transverse processes were clearly visible, and the vertebrae were labeled. Images were then calibrated from pixels to cm using the depth scale on each ultrasound image. The line tool was then used to measure the distances between the tips of the transverse processes of L1/2, L2/3, L3/4, and L4/5, drawing a line from the centre of the first transverse process tip to the centre of the next transverse process tip. The transverse process tip were identified by a bright echo superior to the dark shadow produced by each transverse process (Figure 3) [12]. Forty‐eight images were captured per participant for each visit, with three images captured at each spinal level (L1/2, L2/3, L3/4, and L4/5) and under four loading conditions (Baseline, Unloaded, Loaded 1, and Loaded 2). The mean distance between the transverse processes was recorded for each spinal level and loading condition. The total inter‐transverse process distance was also recorded as the sum of the mean distance between the transverse processes for each spinal level. A second investigator (rater 2) performed blinded measurements between the tips of the labeled transverse processes for each image using Kinovea software as described.

### 2.6 Statistical Analysis

Statistical analyses were performed using SPSS for Windows (SPSS Statistics 28: IBM, Chicago, IL, USA). The normality of the data was confirmed using the Shapiro–Wilk test. Independent *t* tests were used to compare the distances for each spinal level and loading condition between raters, and paired t‐tests were used to compare the distances for each spinal level and loading condition between visits to determine the suitability of the data for pooling. As there were no significant differences between the raters or between the visits, the data from visits 1 and 2 were pooled to assess inter‐rater reliability and agreement for measured distances at each spinal level and loading condition. In addition, the data from raters 1 and 2 were pooled to assess within‐ and between‐visit reliability and agreement for measured distances at each spinal level and loading condition, and the effects of the loading conditions on measured distances at each spinal level. Inter‐rater reliability was calculated using coefficients of variation (CVs) and intraclass correlation coefficients (ICCs) (3,*k*) with 95% upper and lower bound confidence intervals (95% CI) [27]. The guidelines provided by Koo and Li [27] were used to determine the strength of the ICCs, with below 0.5 defined as poor, between 0.5 and 0.75 as moderate, between 0.75 and 0.9 as good, and above 0.9 as excellent. Within‐ and between‐visit reliability was assessed using CV and ICC (3,*k*). Agreement between the raters, and between the visits was assessed using Bland–Altman plots with pooled data from all loading conditions [28]. Acceptable limits of agreement were predefined as ±2 mm for individual spinal levels, based on previous literature reporting limits of agreement ranging between ±1.0 and ±2.3 mm for interspinous and intersegmental distances assessed using ultrasonography [11, 14]. For the total inter‐transverse process distance, a wider acceptable limit of agreement of ±5 mm was defined to account for the cumulative nature of measurement variability across multiple levels [18].

A mixed‐effects one‐way analysis of variance was used to determine the effects of loading condition on measured distances at each spinal level using the pooled data from both raters on both visits. Following significant main effects of loading condition, Dunnett's post hoc tests were used to compare distances at each spinal level between each loading condition. Statistical significance was set at *p* < 0.05. Results are presented as mean ± standard deviation (SD).

## 3 Results

### 3.1 Inter‐Rater Reliability and Agreement

**FIGURE 4:**
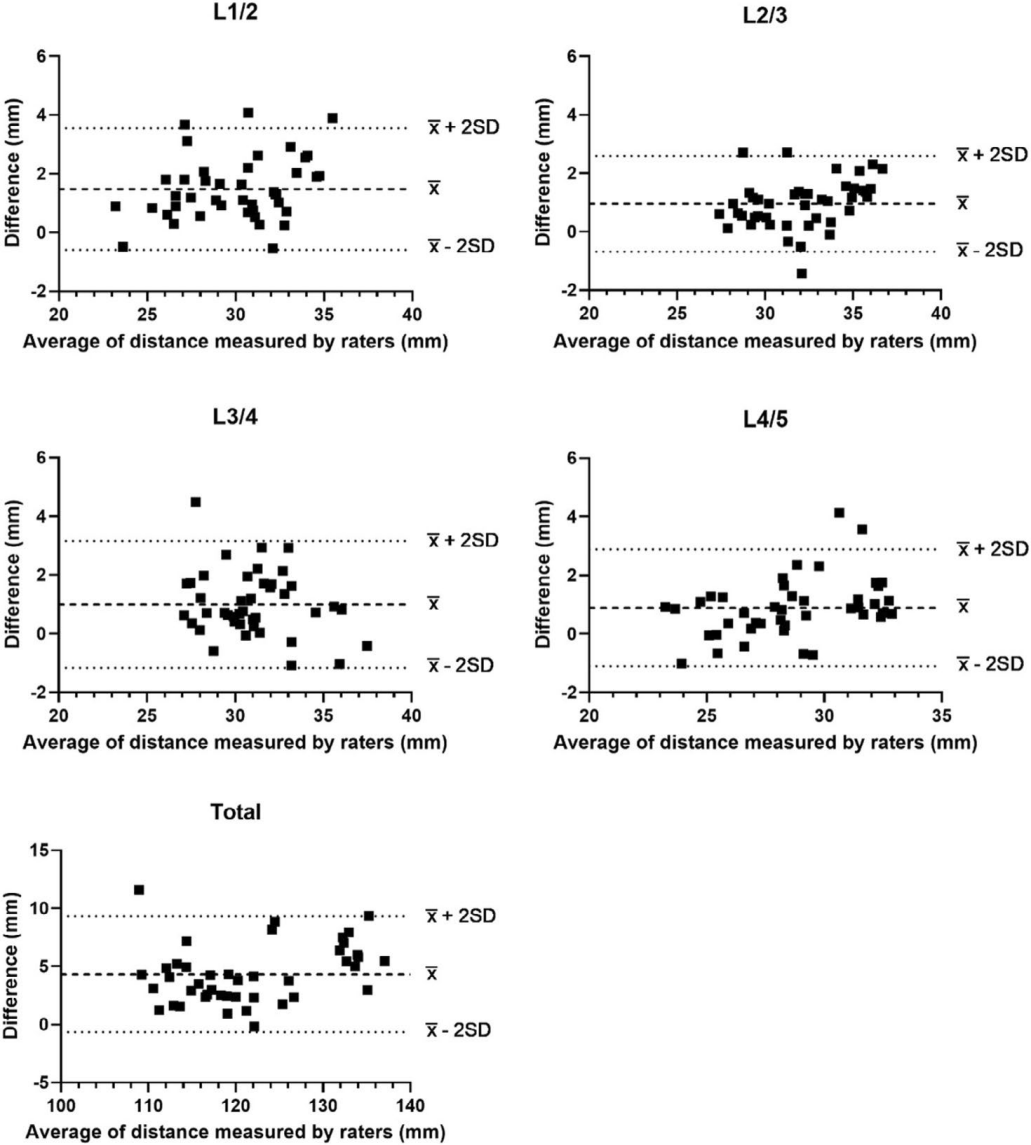
Bland–Altman plots comparing the agreement of the inter‐transverse process distances at each spinal level and total inter‐transverse process distance measured by rater 1 and rater 2. Dashed lines, bias, or mean difference ($\bar{x}$); dotted lines, 95% limits of agreement ($\bar{x}$ ± 2SD).

Inter‐rater reliability was good to excellent with ICCs between 0.81 and 0.99 for all inter‐transverse process distances measured, with no significant differences between raters (Table 1). Inter‐rater CVs were between 6.37% and 12.2% for all measurements. The agreement between rater 1 and rater 2 fell within the predefined acceptable limits of agreement, with a small positive bias by rater 2 (Table 1 and Figure 4).

**TABLE 1:** Mean ± SD (standard deviation) of inter‐transverse process distances at each spinal level and total inter‐transverse process distance for raters 1 and 2; inter‐rater interclass correlation coefficients (ICCs), 95% confidence intervals (CIs), coefficients of variation (CVs), and *p* values; and bias, SD of bias, and 95% limits of agreement (LOA) of Bland–Altman plots comparing raters 1 and 2.

Spinal level	Loading condition	Distance (mm)		Inter‐rater				Bland–Altman plots		
		Rater 1	Rater 2	ICC (3,*k*)	95% CI	CV (%)	*p*	Bias	SD of bias	95% LOA
L1/2	Baseline	29.6 ± 3.46	31.2 ± 3.93	0.96	0.86, 0.99	12.2	0.34	1.48	1.05	−0.59 to 3.54
	Unloaded	29.4 ± 3.00	30.5 ± 3.01	0.99	0.95, 1.0	10.0	0.40			
	Loaded 1	28.7 ± 3.16	30.4 ± 3.51	0.96	0.87, 0.99	11.4	0.26			
	Loaded 2	29.3 ± 2.45	30.9 ± 2.84	0.98	0.94, 1.0	9.01	0.18			
L2/3	Baseline	32.0 ± 2.37	33.1 ± 2.89	0.96	0.86, 0.99	8.11	0.34	0.96	0.84	−0.68 to 2.59
	Unloaded	32.0 ± 2.67	32.9 ± 3.16	0.98	0.93, 1.0	8.91	0.47			
	Loaded 1	28.7 ± 2.92	30.4 ± 2.83	0.99	0.96, 1.0	9.12	0.36			
	Loaded 2	29.3 ± 2.38	30.9 ± 2.54	0.98	0.90, 0.99	7.60	0.58			
L3/4	Baseline	31.7 ± 3.26	32.4 ± 2.91	0.98	0.93, 1.0	9.46	0.61	1.0	1.1	−1.16 to 3.16
	Unloaded	31.2 ± 3.04	32.1 ± 2.44	0.95	0.83, 0.99	8.64	0.43			
	Loaded 1	29.4 ± 1.90	30.5 ± 1.85	0.81	0.30, 0.95	6.37	0.20			
	Loaded 2	29.6 ± 1.79	30.9 ± 2.23	0.95	0.81, 0.99	6.88	0.16			
L4/5	Baseline	29.1 ± 2.54	30.1 ± 2.70	0.98	0.94, 1.0	8.81	0.37	0.89	1.02	−1.1 to 2.88
	Unloaded	28.6 ± 2.57	29.3 ± 3.21	0.95	0.82, 0.99	9.89	0.57			
	Loaded 1	27.5 ± 2.94	28.5 ± 3.35	0.96	0.85, 0.99	11.1	0.46			
	Loaded 2	27.6 ± 2.60	28.4 ± 3.01	0.97	0.89, 0.99	9.89	0.54			
Total distance (L1/5)	Baseline	122 ± 8.96	127 ± 9.81	0.98	0.93, 1.0	7.57	0.29	4.32	2.54	−0.67 to 9.31
	Unloaded	121 ± 8.14	125 ± 8.73	0.99	0.96, 1.0	6.87	0.32			
	Loaded 1	117 ± 7.74	121 ± 8.68	0.96	0.84, 0.99	7.07	0.18			
	Loaded 2	118 ± 6.93	122 ± 8.35	0.97	0.89, 0.99	6.50	0.22			

### 3.2 Within‐ and Between‐Visit Reliability and Agreement

**FIGURE 5:**
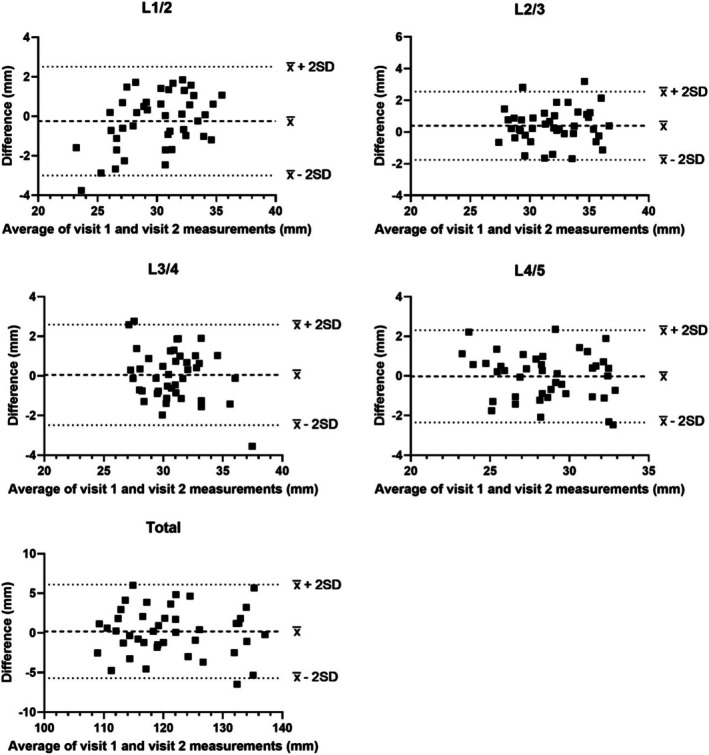
Bland–Altman plots comparing the agreement of inter‐transverse process distances at each spinal level and total inter‐transverse process distance measured on visit 1 and visit 2. Dashed lines represent bias or mean difference ($\bar{x}$); dotted lines represent 95% limits of agreement ($\bar{x}$ ± 2SD).

Within‐visit reliability was excellent with ICCs between 0.91 and 0.99 for all inter‐transverse process distances measured, with no significant differences between visits (Table 2). Within‐visit CVs were between 6.1% and 12.1% for all measurements. Between‐visit reliability was good to excellent with ICCs between 0.87 and 0.98 for all measurements (Table 2). Between‐visit CVs were between 5.24% and 13.0% for all measurements. The agreement between visit 1 and visit 2 fell within the predefined acceptable limits of agreement (Table 2 and Figure 5). There was a random variability in the differences between the measurements at the two visits.

**TABLE 2:** Mean ± SD (standard deviation) of inter‐transverse process distance at each spinal level and total inter‐transverse process distance for visits 1 and 2; within‐ and between‐visit interclass correlation coefficients (ICCs), 95% confidence intervals (CIs), coefficients of variation (CVs), and *p* values; and bias, SD of bias, and 95% limits of agreement (LOA) of Bland–Altman plots comparing visits 1 and 2.

Spinal level	Loading condition	Distance (mm)		Within‐visit			Between‐visit				Bland Altman plots		
		Visit 1	Visit 2	ICC (3,*k*)	95% CI	CV (%)	ICC (3,*k*)	95% CI	CV (%)	*p*	Bias	SD of bias	95% LOA
L1/2	Baseline	30.5 ± 3.43	30.3 ± 3.91	0.96	0.92, 0.99	12.1	0.97	0.90, 0.99	11.8	0.69	−0.24	1.41	−3.0 to 2.52
	Unloaded	29.8 ± 2.85	30.0 ± 3.38	0.97	0.93, 0.99	10.4	0.95	0.79, 0.99	10.2	0.90			
	Loaded 1	29.9 ± 2.91	29.2 ± 3.79	0.96	0.89, 0.98	11.4	0.94	0.77, 0.98	11.2	0.15			
	Loaded 2	30.1 ± 2.60	30.1 ± 2.84	0.93	0.81, 0.98	9.0	0.93	0.72, 0.99	8.80	0.88			
L2/3	Baseline	32.4 ± 2.61	32.8 ± 2.76	0.96	0.91, 0.98	8.3	0.93	0.73, 0.98	8.08	0.40	0.40	1.10	−1.75 to 2.55
	Unloaded	31.8 ± 2.87	32.7 ± 2.89	0.98	0.96, 0.99	8.9	0.98	0.91, 0.99	8.80	0.07			
	Loaded 1	31.3 ± 2.84	31.6 ± 2.98	0.97	0.94, 0.99	9.2	0.97	0.87, 0.99	9.04	0.40			
	Loaded 2	31.6 ± 2.46	32.0 ± 2.49	0.95	0.89, 0.99	7.8	0.95	0.79, 0.99	7.61	0.30			
L3/4	Baseline	32.3 ± 3.38	31.9 ± 2.88	0.95	0.91, 0.98	9.7	0.95	0.81, 0.99	9.57	0.36	0.05	1.30	−2.49 to 2.59
	Unloaded	31.4 ± 2.50	31.5 ± 2.76	0.94	0.88, 0.98	8.4	0.95	0.78, 0.99	8.18	0.37			
	Loaded 1	29.7 ± 2.05	30.2 ± 1.58	0.92	0.84, 0.97	6.1	0.87	0.52, 0.97	6.01	0.25			
	Loaded 2	30.0 ± 2.03	30.5 ± 2.14	0.91	0.78, 0.97	6.9	0.89	0.55, 0.97	6.75	0.28			
L4/5	Baseline	29.9 ± 2.76	29.3 ± 2.52	0.95	0.87, 0.99	8.9	0.97	0.87, 0.99	8.76	0.10	−0.02	1.19	−2.35 to 2.31
	Unloaded	28.7 ± 2.77	29.0 ± 2.98	0.99	0.97, 0.99	9.9	0.98	0.91, 0.99	9.74	0.88			
	Loaded 1	27.8 ± 3.50	28.2 ± 2.82	0.99	0.98, 1.0	11.3	0.94	0.78, 0.98	11.1	0.44			
	Loaded 2	28.0 ± 2.75	28.0 ± 2.94	0.96	0.84, 1.0	10.2	0.95	0.80, 0.99	9.90	0.91			
Total distance (L1/5)	Baseline	125 ± 9.25	124 ± 9.66	0.94	0.63, 1.0	7.6	0.97	0.88, 0.99	7.41	0.50	0.19	3.01	−5.71 to 6.09
	Unloaded	122 ± 8.06	123 ± 8.50	0.98	0.94, 1.0	6.8	0.98	0.90, 0.99	6.62	0.33			
	Loaded 1	119 ± 8.49	119 ± 8.05	0.96	0.86, 0.99	7.0	0.94	0.79, 0.99	6.79	0.78			
	Loaded 2	120 ± 7.75	121 ± 7.58	0.94	0.80, 0.99	6.4	0.98	0.91, 0.99	6.22	0.27			

### 3.3 Effects of Loading Condition

**FIGURE 6:**
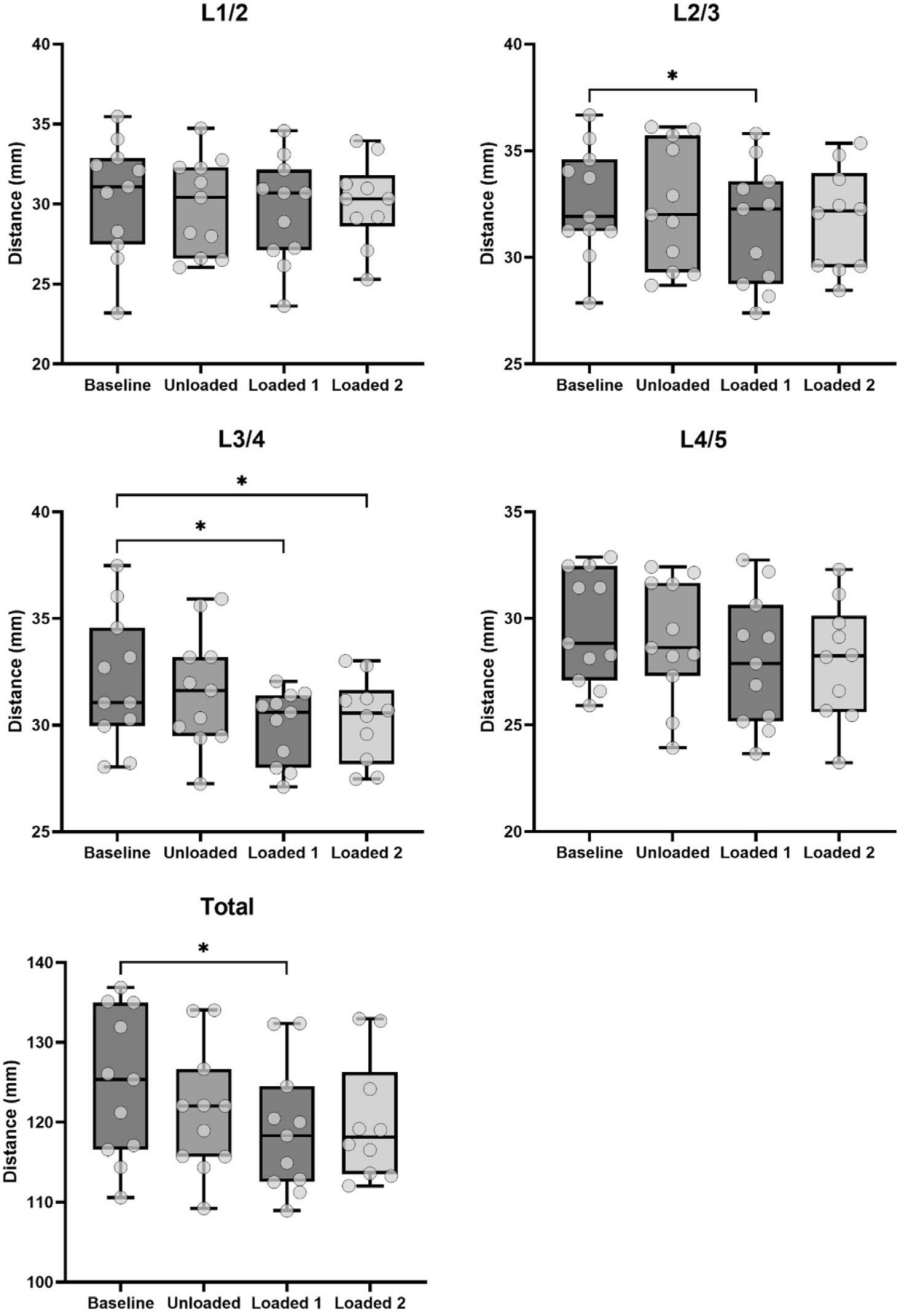
Mean ± standard deviation of inter‐transverse process distances at each spinal level and total inter‐transverse process distance for each loading condition. *Significant difference from baseline (*p* < 0.05).

There was no main effect of loading condition on inter‐transverse process distances for spinal level L1/2 (*F*_1,18_ = 0.67; *p* = 0.52) and L4/5 (*F*_1,11_ = 3.82; *p* = 0.07) (Figure 6). There were significant main effects of loading condition for inter‐transverse process distance L2/3 (*F*_2,24_ = 5.44; *p* = 0.01), L3/4 (*F*_1,12_ = 9.26; *p* = 0.01), and total distance (*F*_2,20_ = 4.64; *p* = 0.02). L3/4 inter‐transverse process distances decreased from Baseline to Loaded 1 (*p* = 0.02) and Loaded 2 (*p* = 0.02), with no pairwise differences between Baseline to Unloaded (*p* = 0.32). L2/3 inter‐transverse process distances decreased from Baseline to Loaded 1 (*p* = 0.03), with no pairwise differences between Baseline to Unloaded (*p* = 0.97) or Loaded 2 (*p* = 0.10). Total inter‐transverse process distance decreased from Baseline to Loaded 1 (*p* = 0.03), with no pairwise differences between Baseline to Unloaded (*p* = 0.25) or Loaded 2 (*p* = 0.08).

## 4 Discussion

### 4.1 Main Findings

We assessed the inter‐rater and within‐ and between‐visit reliability of ultrasonography to quantify spinal compression via measurement of lumbar inter‐transverse process distance in healthy participants while standing unloaded, and after 15 and 30 min of heavy load carriage. We found good to excellent inter‐rater reliability with good agreement between raters, as well as good to excellent within‐ and between‐visit reliability with good agreement between visits. Furthermore, this ultrasonography method was able to detect significant spinal compression at levels L2/3, L3/4, and total inter‐transverse process distance during standing with heavy load carriage.

### 4.2 Inter‐Rater Reliability and Agreement

We found no significant differences in the measurement of inter‐transverse process distance between raters, and good to excellent inter‐rater reliability. The range of ICCs across our measurements (0.81–0.99) sits towards the top end of the range of ICCs reported previously (0.61–0.99) in studies using ultrasonography to measure lumbar inter‐segmental distances [13, 14, 15, 16]. In addition, we found good agreement between the measurements of the raters, falling within the predefined acceptable limits of agreement. A positive bias was observed, with rater 2 tending to measure slightly higher values on average. These findings are comparable to a similar study investigating agreement between raters measuring inter‐spinous process distances with participants lying prone, on elbows, or kneeling [16]. The presence of a bias in the present study may be due to rater 2 generally measuring higher on the tips of transverse processes than rater 1. However, there were no significant differences between raters, and the level of compression found was consistent between raters. To improve consistency between raters, additional pre‐testing side‐by‐side ultrasound analyses could be undertaken between the raters. These results suggest that the inter‐rater reliability and agreement of the methods employed in the present study are comparable to similar studies that used different lumbar vertebral landmarks and were not performed on standing participants or during heavy load carriage. Our findings suggest that ultrasonography measurement of the inter‐transverse process distances in the lumbar vertebra can be reliably undertaken with good agreement between two raters during standing heavy load carriage.

### 4.3 Within‐ and Between‐Visit Reliability and Agreement

We found excellent within‐visit reliability with ICCs between 0.91 and 0.99, and CVs between 6.1% and 12.1%. These results are similar to those found in other studies utilizing ultrasound to measure distances between different lumbar vertebral landmarks in participants lying supine or prone, on elbows, or kneeling [11, 12, 13, 14, 15, 16]. Between‐visit reliability in the present study was good to excellent, with ICC values between 0.87 and 0.98, and CVs between 6.01% and 11.8%, and high agreement between measurements taken at each visit. Our results are similar to those of Tozawa et al. [14], who found the between‐visit ICCs for interspinous process distances measured by ultrasonography to be over 0.99. The use of a standing platform to ensure consistent body position between measurements may have contributed to the similar reliability of our study to those undertaking measurements on prone or supine participants [11, 12, 13, 14, 15, 16]. Our findings suggest that measurement of the inter‐transverse process distances in the lumbar vertebra with ultrasonography using these methods during standing heavy load carriage is reliable within and between visits.

### 4.4 Effects of Loading Condition

We observed a significant reduction in inter‐transverse process distance at levels L2/3 and L3/4, and across the total inter‐transverse process distance following 15 and 30 min of standing with a heavy load of 25 kg. There were no significant changes at levels L1/2 or L4/5. Our results are consistent with other studies utilizing ultrasonography, which found a reduction in total lumbar spine height following loaded sitting [18], and maximal displacement occurring between L2 and L4 in cadaveric lumbar spines placed under traction [13]. Contrasting these findings, lumbar intervertebral distances at L3/4 and L4/5 measured via transabdominal ultrasound in supine, standing, and loaded standing conditions, decreased at L4/5 but not at L3/4 [17]. This may be due to imaging from an anterior rather than posterior position, as MRI imaging shows that the effect of load on intervertebral disc heights differs anteriorly and posteriorly [19]. Our results supports prior work that has observed differing responses to loading at each lumbar level. [13, 17, 19] For example, research utilizing upright MRI reported that heavy loads increased lordosis at the superior lumbar levels and kyphosis at the inferior lumbar levels, with a transition at L3/4, which was dependent on the presence of a load [19]. This highlights the importance of considering changes to inter‐vertebral joint angles which occur during loading [19] and how these might affect inter‐process distances depending on the imaging position and spinal level when interpreting results. Nevertheless, the load‐related reduction in inter‐transverse process distance found in the present study is consistent with the literature showing reductions in inter‐vertebral distance resulting from occupational loads [19, 29], which are associated with an increased risk of back pain and spinal injury [30, 31, 32].

### 4.5 Limitations and Future Directions

A limitation of this study was that the cohort consisted of a relatively small sample of healthy, working‐age adults. The generalizability of these results to clinical or occupational populations with pre‐existing spinal pathology, altered spinal mechanics, or chronic exposure to load carriage should be confirmed. In addition, assessments were limited to static standing conditions. In practice, operational scenarios often involve heavier, unevenly distributed loads carried dynamically for prolonged periods, and these differences should be considered when interpreting the present findings. Future studies should explore the feasibility and reliability of ultrasonographic assessment during dynamic load carriage, including walking, running, or field operations under load, where motion artifact, probe stability, and anatomical access may pose additional challenges. Innovations in wearable ultrasound or stabilization techniques may further support dynamic imaging in future investigations.

## 5 Conclusion

We used ultrasonography to assess spinal compression during standing heavy load carriage via measurement of inter‐transverse process distances in the lumbar vertebra and found that this was reliable between raters, within and between visits, and with a good level of agreement between the two raters and two visits. In addition, we found that ultrasonography was able to quantify spinal compression at L2/3, L3/4, and total lumbar spinal distance following heavy load carriage. The methods used in this study could provide a practical and affordable method of reliably assessing changes in spinal compression during heavy load carriage which may be used to assess the results of interventions designed to reduce spinal compression during heavy load carriage.

## Conflicts of Interest

The authors declare no conflicts of interest.

## Data Availability

The data that support the findings of this study are available from the corresponding author upon reasonable request.

## References

[1] GBD 2021 Low Back Pain Collaborators , “Global, Regional, and National Burden of Low Back Pain, 1990‐2020, Its Attributable Risk Factors, and Projections to 2050: A Systematic Analysis of the Global Burden of Disease Study 2021,” Lancet Rheumatology 5, no. 6 (2023): e316–e329. 37273833 10.1016/S2665-9913(23)00098-X PMC10234592

[2] M. A. Adams and P. Dolan , “Intervertebral Disc Degeneration: Evidence for Two Distinct Phenotypes,” Journal of Anatomy 221, no. 6 (2012): 497–506. 22881295 10.1111/j.1469-7580.2012.01551.x PMC3512277

[3] M. J. DePalma , J. M. Ketchum , and T. Saullo , “What Is the Source of Chronic Low Back Pain and Does Age Play a Role?,” Pain Medicine 12, no. 2 (2011): 224–233. 21266006 10.1111/j.1526-4637.2010.01045.x

[4] P. Kjaer , C. Leboeuf‐Yde , L. Korsholm , J. S. Sorensen , and T. Bendix , “Magnetic Resonance Imaging and Low Back Pain in Adults: A Diagnostic Imaging Study of 40‐Year‐Old Men and Women,” Spine 30, no. 10 (2005): 1173–1180. 15897832 10.1097/01.brs.0000162396.97739.76

[5] L. G. Macedo and M. C. Battie , “The Association Between Occupational Loading and Spine Degeneration on Imaging ‐ a Systematic Review and Meta‐Analysis,” BMC Musculoskeletal Disorders 20, no. 1 (2019): 489. 31656182 10.1186/s12891-019-2835-2 PMC6815427

[6] D. J. Schofield , R. N. Shrestha , M. Cunich , et al., “Lost Productive Life Years Caused by Chronic Conditions in Australians Aged 45–64 Years, 2010–2030,” Medical Journal of Australia 203, no. 6 (2015): 260. 10.5694/mja15.00132 26377293

[7] R. Orr , V. Johnston , J. Coyle , R. M. Orr , and R. Pope , “Reported Load Carriage Injuries of the Australian Army Soldier,” Journal of Occupational Rehabilitation 25, no. 2 (2015): 316–322. 25178432 10.1007/s10926-014-9540-7

[8] M. Hupli , R. Heinonen , and H. Vanharanta , “Height Changes Among Chronic Low Back Pain Patients During Intense Physical Exercise,” Scandinavian Journal of Medicine & Science in Sports 7, no. 1 (1997): 32–37. 9089902 10.1111/j.1600-0838.1997.tb00114.x

[9] I. A. F. Stokes , M. G. Gardner‐Morse , and S. M. Henry , “Intra‐Abdominal Pressure and Abdominal Wall Muscular Function: Spinal Unloading Mechanism,” Clinical Biomechanics 25, no. 9 (2010): 859–866. 20655636 10.1016/j.clinbiomech.2010.06.018 PMC2949466

[10] A. Adams , O. Roche , A. Mazumder , I. Davagnanam , and K. Mankad , “Imaging of Degenerative Lumbar Intervertebral Discs; Linking Anatomy, Pathology and Imaging,” Postgraduate Medical Journal 90, no. 1067 (2014): 511–519. 24965489 10.1136/postgradmedj-2013-132193

[11] G. S. Chleboun , M. J. Amway , J. G. Hill , K. J. Root , H. C. Murray , and A. V. Sergeev , “Measurement of Segmental Lumbar Spine Flexion and Extension Using Ultrasound Imaging,” Journal of Orthopaedic and Sports Physical Therapy 42, no. 10 (2012): 880–885. 22814284 10.2519/jospt.2012.3915

[12] J. R. Ledsome , V. Lessoway , L. E. Susak , F. A. Gagnon , R. Gagnon , and P. C. Wing , “Diurnal Changes in Lumbar Intervertebral Distance, Measured Using Ultrasound,” Spine 21, no. 14 (1996): 1671–1675. 8839471 10.1097/00007632-199607150-00012

[13] S. Sobczak , P. M. Dugailly , K. K. Gilbert , et al., “Reliability and Validation of In Vitro Lumbar Spine Height Measurements Using Musculoskeletal Ultrasound: A Preliminary Investigation,” Journal of Back and Musculoskeletal Rehabilitation 29, no. 1 (2016): 171–182. 26406194 10.3233/BMR-150613

[14] R. Tozawa , M. Katoh , H. Aramaki , et al., “Absolute and Relative Reliability of Lumbar Interspinous Process Ultrasound Imaging Measurements,” Journal of Physical Therapy Science 28, no. 8 (2016): 2210–2213. 27630399 10.1589/jpts.28.2210 PMC5011563

[15] R. Tozawa , M. Katoh , H. Aramaki , T. Kumamoto , and O. Fujinawa , “Reliability and Validity of an Ultrasound‐Based Imaging Method for Measuring Interspinous Process Distance in the Lumbar Spine Using Two Different Index Points,” Journal of Physical Therapy Science 27, no. 7 (2015): 2333–2336. 26311976 10.1589/jpts.27.2333 PMC4540874

[16] R. Tozawa , M. Katoh , T. Kawasaki , H. Aramaki , T. Kumamoto , and O. Fujinawa , “Reliability of Ultrasound to Measure the Distance Between Lumbar Interspinous Processes,” Medical Engineering & Physics 99 (2022): 103740. 35058022 10.1016/j.medengphy.2021.103740

[17] N. Ulmi , N. Wiesmann , M. Egli , J. Swanenburg , and R. Sutter , “Impact of Posture and Axial Loading on Lumbar Intervertebral Disc Dimensions Investigated by Transabdominal Ultrasound,” European Journal of Radiology 181 (2024): 111729. 39260210 10.1016/j.ejrad.2024.111729

[18] V. Poortmans , J. M. Brismee , B. Poortmans , et al., “Assessment of Lumbar Spine Height Following Sustained Lumbar Extension Posture: Comparison Between Musculoskeletal Ultrasonography and Stadiometry,” Journal of Manipulative and Physiological Therapeutics 39, no. 8 (2016): 586–593. 27637322 10.1016/j.jmpt.2016.07.003

[19] A. E. Rodríguez‐Soto , R. Jaworski , A. Jensen , et al., “Effect of Load Carriage on Lumbar Spine Kinematics,” Spine 38, no. 13 (2013): E783–E791. 23524870 10.1097/BRS.0b013e3182913e9f

[20] S. C. Chan , S. J. Ferguson , and B. Gantenbein‐Ritter , “The Effects of Dynamic Loading on the Intervertebral Disc,” European Spine Journal 20, no. 11 (2011): 1796–1812. 21541667 10.1007/s00586-011-1827-1 PMC3207351

[21] C. Liu , J. Ran , J. N. Morelli , B. Hou , Y. Li , and X. Li , “Determinants of Diurnal Variation in Lumbar Intervertebral Discs and Paraspinal Muscles: A Prospective Quantitative Magnetic Resonance Imaging Study,” European Journal of Radiology 160 (2023): 110712. 36720179 10.1016/j.ejrad.2023.110712

[22] Exercise and Sports Science Australia , “Fitness Australia, Sports Medicine Australia, Adult Pre‐Exercise Screening System,” 2021, https://www.essa.org.au/Public/ABOUT_ESSA/Pre‐Exercise_Screening_Systems.aspx.

[23] S. Shymon , A. R. Hargens , L. A. Minkoff , and D. G. Chang , “Body Posture and Backpack Loading: An Upright Magnetic Resonance Imaging Study of the Adult Lumbar Spine,” European Spine Journal 23, no. 7 (2014): 1407–1413. 24619606 10.1007/s00586-014-3247-5 PMC6339990

[24] R. M. Orr , R. Pope , V. Johnston , and J. Coyle , “Soldier Occupational Load Carriage: A Narrative Review of Associated Injuries,” International Journal of Injury Control and Safety Promotion 21, no. 4 (2014): 388–396. 24028439 10.1080/17457300.2013.833944

[25] D. Z. Nin , M. T. G. Pain , Y. H. Lim , et al., “Hamstring Muscle Architecture and Viscoelastic Properties: Reliability and Retrospective Comparison Between Previously Injured and Uninjured Athletes,” Journal of Mechanics in Medicine and Biology 21, no. 1 (2021): 2150007.

[26] S. Telfer , J. Woodburn , and D. E. Turner , “An Ultrasound Based Non‐Invasive Method for the Measurement of Intrinsic Foot Kinematics During Gait,” Journal of Biomechanics 47, no. 5 (2014): 1225–1228. 24433670 10.1016/j.jbiomech.2013.12.014

[27] T. K. Koo and M. Y. Li , “A Guideline of Selecting and Reporting Intraclass Correlation Coefficients for Reliability Research,” Journal of Chiropractic Medicine 15, no. 2 (2016): 155–163. 27330520 10.1016/j.jcm.2016.02.012 PMC4913118

[28] J. M. Bland and D. G. Altman , “Statistical Methods for Assessing Agreement Between Two Methods of Clinical Measurement,” Lancet 1, no. 8476 (1986): 307–310. 2868172

[29] P. Brinckmann , W. Frobin , M. Biggemann , et al., “Quantification of Overload Injuries to Thoracolumbar Vertebrae and Discs in Persons Exposed to Heavy Physical Exertions or Vibration at the Workplace Part II Occurrence and Magnitude of Overload Injury in Exposed Cohorts,” Clinical Biomechanics 13, no. 2 (1998): S1–S36. 10.1016/s0268-0033(98)00050-3 11430793

[30] R. B. Dunlop , M. A. Adams , and W. C. Hutton , “Disc Space Narrowing and the Lumbar Facet Joints,” Journal of Bone and Joint Surgery. British Volume 66, no. 5 (1984): 706–710. 6501365 10.1302/0301-620X.66B5.6501365

[31] A. Seidler , A. Bergmann , M. Jäger , et al., “Cumulative Occupational Lumbar Load and Lumbar Disc Disease—Results of a German Multi‐Center Case‐Control Study (EPILIFT),” BMC Musculoskeletal Disorders 10 (2009): 48. 19422710 10.1186/1471-2474-10-48 PMC2689164

[32] I. A. Stokes and J. C. Iatridis , “Mechanical Conditions That Accelerate Intervertebral Disc Degeneration: Overload Versus Immobilization,” Spine 29, no. 23 (2004): 2724–2732. 15564921 10.1097/01.brs.0000146049.52152.da PMC7173624

